# Emphasizing Pulmonary Function With Respiratory Therapy in an Infant With Congenital Adrenal Hyperplasia, Bronchopneumonia and Leptomeningitis

**DOI:** 10.7759/cureus.28679

**Published:** 2022-09-01

**Authors:** Pratik R Jaiswal, Pallavi Bhakaney, Vaishnavi Yadav, Moli Jain, Vishnu Vardhan

**Affiliations:** 1 Department of Cardiorespiratory Physiotherapy, Ravi Nair Physiotherapy College, Datta Meghe Institute of Medical Sciences (DMIMS), Wardha, IND

**Keywords:** respiratory physiotherapy, pulmonary function, bronchopneumonia, leptomeningitis, congenital adrenal hyperplasia, adrenal crisis

## Abstract

A set of hereditary diseases affecting the adrenal glands, a couple of walnut-sized structures above the kidneys, is known as congenital adrenal hyperplasia (CAH). The adrenal glands generate essential hormones like cortisol, mineralocorticoids, and androgens. It is usually diagnosed in the early neonatal period. The documentation of this case study is aimed to provide a case of an infant with CAH associated with bronchopneumonia and leptomeningitis. Brought with complaints of poor feeding, moderate fever associated with chills, and loose stools, was an 11-months-old patient with a known diagnosis of CAH. Symptoms have shown that the baby was in a possible adrenal crisis. He was intubated with an endotracheal tube after repeated episodes of seizures. Investigations revealed signs of bronchopneumonia and leptomeningitis. A thorough assessment, once completed, helped in analyzing relevant problems to be treated with respiratory therapy. Respiratory therapy, in this case, was aimed at improving the blood oxygen levels and assisting breathing by clearing out secretions and opening up the airways. Parent education was the foremost part of the treatment regime, followed by bronchodilator administration, conventional chest physiotherapy, postural drainage positions in the mother’s lap, lung squeezing technique, and perioral pressure. A holistic and multidisciplinary approach is beneficial in patients with CAH undergoing an adrenal crisis. It needs to be offered to the patients who would be benefited, considering the effect, which needs to be reassessed on specific outcomes with changes in the management with the improving condition.

## Introduction

Congenital adrenal hyperplasia (CAH) is an autosomal recessive condition disrupting the steroid synthesis pathway, resulting in adrenal inadequacy at a calculated incidence rate of one in 15,000 live births. It is caused by a genetically acquired impairment of enzymes engaged in the production of glucocorticoids and mineralocorticoids. It is usually detected using a neonatal metabolic testing panel in the initial neonatal period [1]. 21-hydroxylase deficiency (21OHD, CYP21A2 mutation) represents the most prevalent enzyme deficit (90%-99%) causing CAH, with a frequency of about 1 in 15,000, although being more prevalent in some racial backgrounds. Non-classic (NC) CAH is more common and can affect up to 1:200 people of different racial and cultural backgrounds [2]. 21-hydroxylase insufficiency is divided into two categories: classic (serious phenotype) and non-classic (less serious phenotype). The simple-virilizing (SV) and salt-wasting (SW) subtypes of the classic phenotype are subsequently classified depending on either aldosterone generation is sufficient or insufficient, accordingly [3]. Small kids and newborns are frequently affected by the communicable disease bronchopneumonia. Mycoplasma pneumonia is the principal reason for bronchopneumonia, but "combined illnesses" driven by viruses and microbes have also been documented [4]. A correct and prompt diagnosis, as well as the characterization of the pathogen, are essential since encephalitis is a severe clinical illness with substantial morbidity and mortality [5]. Unlike sarcoids, which are normally well-formed, non-necrotic, and do not have a significant lymphoplasmacytic inflammatory element, leptomeningeal granulomas were different from these characteristics [6]. While leptomeningeal participation is more frequently associated with mental competency changes, hemiparesis, and seizures, dural participation is more frequently associated with migraines and nerve palsy [7]. Cerebrospinal fluid antibodies against the GluN1 subunit of NMDAR are present in anti-NMDAR encephalitis, an immune-mediated condition that is characterized by complicated neuropsychiatric symptoms. It has been discovered to be the most dominant kind of autoimmune encephalitis since its initial discovery in 2007 [8]. Bronchopneumonia in grownups is commonly treated with an IV injection of ambroxol hydrochloride [9]. Pediatric meningitis is characterized by interchanging clinical characteristics like temperature, pleocytosis of CSF, and encephalitis, with added results of encephalopathy, focal neurologic findings, seizures, and/or abnormal neuroimaging/electroencephalography, that are classically non-specific to the primary etiology [10].

## Case presentation

Patient information

The parents of an 11-month-old boy with CAH took him to the Pediatrics Outpatient department (OPD) with complaints of mild graded fever, chills, loose stools, low food intake, and potential agitation on micturition during the previous three to four days. The baby was approached to a local hospital with the same concerns, and antibiotics were given, according to the parents. However, the baby was still irritable, and the intake of food continued to be poor, hence was taken to our tertiary care hospital. A detailed history of the parents revealed that they had consanguineous marriage and their older child (aged three years) also suffers from CAH; which was diagnosed in the neonatal period itself, and takes tablets of hydrocortisone and Tab. Flu-cortisone. In addition, the mother stated that her second child was examined for CAH screening after birth and was confined to the neonatal ICU for roughly seven days. The baby was advised to be fed orally upon admission, and injection of ceftriaxone, Inj. Amikacin and Tab. hydrocortisone were commenced. On the same day, the child's engagement was excellent. The baby had three instances of generalized tonic-clonic seizures the next day, over which Inj. sodium valproate was given, following Inj. midazolam. Following the infusion of the medications, the baby became stable. The baby had another seizure on the same day in the evening that persisted for more than five minutes and was not terminated with Inj. midazolam. On synchronized intermittent mandatory ventilation (SIMV) mode, the baby was voluntarily intubated with an endotracheal tube. The baby was kept under monitoring in case he developed another seizure. Although there was no evidence of a seizure, the baby got rashes all over his body. A dermatologist's advice was sought, and many hyperpigmented macules were discovered all across the body. Considering the baby's current state, required tests were performed, including an MRI brain scan, blood culture, and scrub typhus screening. After that, the baby was confirmed with leptomeningitis. Extubation was scheduled after five days as the baby's condition improved. A physiotherapy call was made after extubation due to increasing tracheobronchial secretions.

Clinical findings

The baby was found to be in a supine laying posture with a well-secured head and neck thanks to the use of a pillow. The patient's eyes were fixated on one side, which the therapist observed. There was labored breathing; Ryle's tube and a central line were in situ; nasal prongs were connected to 5 liters of O2. The newborn was around 66 cm tall and weighed 7 kg. He was afebrile but appeared to be sluggish. Vital signs appeared as follows: Heart rate of 36 beats/minute, respiratory rate of 58 breaths/minute, blood pressure of 94/58 mmHg, and SpO2 was 99%. Auscultation revealed the presence of bilateral conducting sounds.

Diagnostic assessment

**Figure 1:**
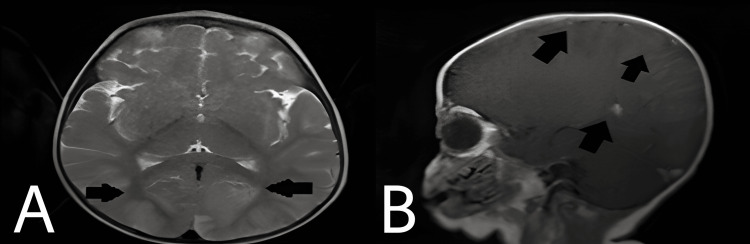
MRI of the brain shows symmetrical moderate-enhancing altered signal intensity cortex of right frontal and bilateral temporo-parieto-occipital regions (A) and in the splenium of corpus callosum (B).

**Figure 2:**
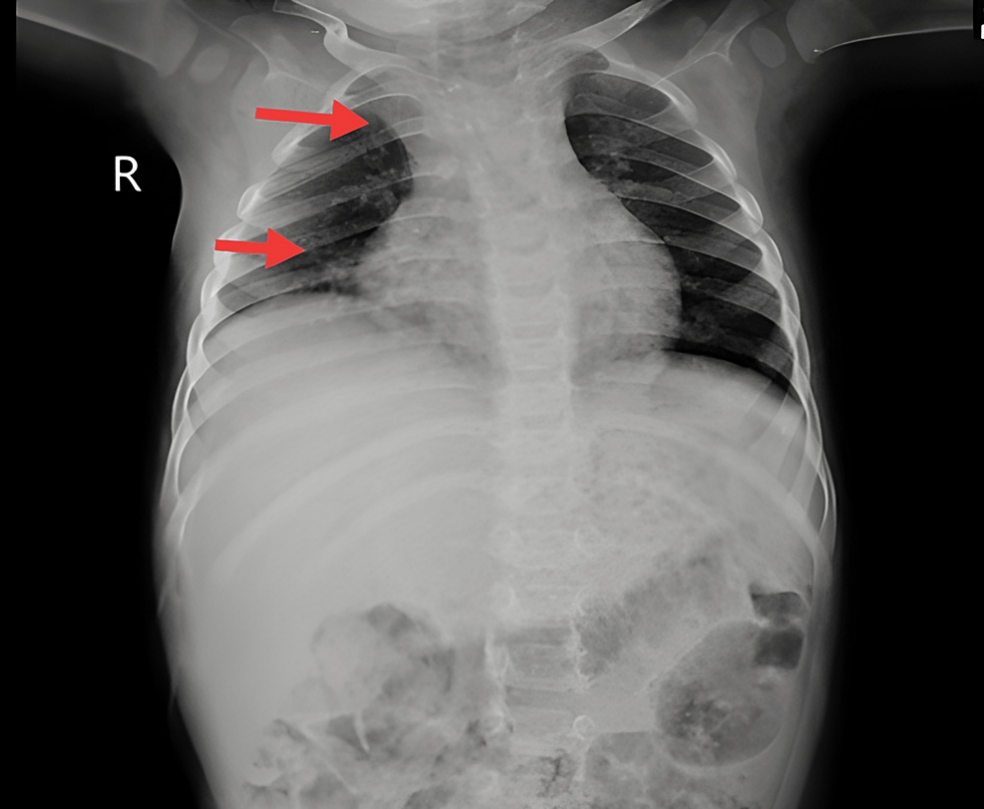
Chest X-ray of the patient featured broncho-pulmonary markings evident of infection and widening of mediastinum.

Laboratory investigations were done, which showed decreased hemoglobin content (6.2 g/dL), increased calcium (6.4 mmol/L), decreased total platelet count (0.65), and potassium (2.8 mmol/L). Radiological investigations included MRI brain, which showed symmetrical moderate-enhancing altered signal intensity cortex of right frontal and bilateral temporo-parieto-occipital regions and in the splenium of corpus callosum significant to viral encephalitis with leptomeningitis (Figure 1). In addition, a chest X-ray showed marked broncho-pulmonary markings evident of infection and widening of mediastinum (Figure 2).

Therapeutic interventions

The physiotherapist's main goal was to clear tracheobronchial secretions, which decreased airway resistance and airway obstruction, leading to better air circulation. As an outcome, the respiratory muscle pump acts better effectively, lowering the amount of effort required to breathe. The physical therapist attended to see the child twice a day for 20-25 minutes per session. Before the procedure, the patient's intracranial and cerebral perfusion pressures were investigated thoroughly. Before the treatment program progressed, the child's mother was instructed on the great significance of physiotherapy. Prior to chest rehabilitation, a bronchodilator was delivered by nebulization for 5-10 minutes. Manual percussions of the thoracic wall were performed bilaterally with a percussor cup, accompanied by mechanical chest vibrations less than 60 Hz. These approaches benefited in moving mucus and secretions forward to the larger airways, making coughing better. The approaches were not administered too rapidly considering the patient's age and related stiff and non-compliant lungs. Across both sides, body positioning was performed every two hours to permit gravity to assist in mobilizing secretions from the anterior, lateral, superior, basal, and posterior regions. In an attempt to clear different lung lobes, the mother was trained postural drainage methods on her lap in supine, prone, and side-lying positions. To sustain a comfortable gravity-assisted position, the patient was further assisted with cushions and small towel rolls. Every posture was sustained for two to five minutes. Coughing was induced via tracheal stimulation. The lung squeezing technique was adopted to improve the uniform distribution of airflow throughout the lungs. The newborn was lying down, and the therapist positioned one hand on the postero-lateral side of one hemithorax and the other on the infant's anterior chest, which extended from the lower ribs to the clavicle. First, three to four total chest compressions spanning five seconds each were delivered. On the opposite side, the same approach was used. In addition, for five seconds, perioral pressure was put on the patient's upper lip. To encourage deep breathing, this exercise should be repeated five times. In the prone position, manual neck stimulations were provided to encourage neck extension. Neck stability and rolling beginning were also achieved through ball exercises. As per Rood's method, the icing was applied to the neck extensors. Following an improvement in the patient's general state, he was sent to neuro-rehabilitation for successful treatment of the delayed developmental milestones.

Patient perspective

The patient's parents were highly cooperative and happy throughout the sessions. They said, "Our baby has shown an improvement regarding his breathing difficulty, which was present at the time we came here, with the help of physiotherapy treatment." They were happy with their child's progress and eager to keep going because the child appreciated the sessions.

Follow-up and outcome of interventions

Outcome measure scores from the first, second, and third weeks are shown in Table 1.

**Table 1:** The effectiveness of the treatment regime after intervention evaluated using outcome measures. E: Eye opening; V: Verbal response; M: Motor response.

Scale	Week 1	Week 2	Week 3
Pediatric Glasgow Coma Scale	E_4_V_4_M_3_	E_4_V_4_M_4_	E_4_V_5_M_5_
Kristjansson Respiratory Score	3	1	0
Functional Status Scale			
Mental Status	Moderate Dysfunction	Mild Dysfunction	Mild Dysfunction
Sensory	Severe Dysfunction	Moderate Dysfunction	Moderate Dysfunction
Communication	Severe Dysfunction	Moderate Dysfunction	Mild Dysfunction
Motor Function	Severe Dysfunction	Severe Dysfunction	Severe Dysfunction
Feeding	Severe Dysfunction	Moderate Dysfunction	Normal
Respiratory	Severe Dysfunction	Mild Dysfunction	Normal

## Discussion

The most frequent variety of CAH is a 21-hydroxylase deficiency, produced by transmutations or removals in the CYP21A gene. It accounts for more than 90% of cases. CAH is a condition that affects the production of adrenal hormones. Adrenal hormones, among the modulator chemicals, have been hypothesized to have essential regulatory and trophic effects on central nervous system cell survival, differentiation, maturation, and synaptogenesis [1]. Glucocorticoid (GC) programs that strike the right ratio among adequate growth and maturation while limiting hyperandrogenism and the potential for undesirable outcomes attributed to adrenal insufficiency are required for the best care of children with CAH [11]. A human's immune system is influenced by his age. Because a child's immunity system is still underdeveloped from the first to the fifth year, the younger they are, the more likely they are to become sick. Infection risk is also less in children under the age of five than in older children. Children aged 13-28 months are the most common victims of pneumonia. Because of the physical differences in the respiratory system structure between males and females, male children are more likely to get pneumonia. Boys' respiratory tracts are generally smaller than girls' in terms of size. Many variables contribute to the male being more susceptible to infective disease, including hormonal variations, environmental exposure, parental disparities, and the immune response in boys [12].

## Conclusions

Respiratory therapy has led to show an overall improvement in the lung function of an infant with CAH. A multidisciplinary approach definitely improved the patient's overall health condition and contributed to a better quality of life. However, studies are required to foreground the effectiveness of rehabilitation in patients with CAH.
